# Improved Predictability of Microstructure Evolution during Hot Deformation of Titanium Alloys

**DOI:** 10.3390/ma13245678

**Published:** 2020-12-12

**Authors:** Ricardo Henrique Buzolin, Franz Miller Branco Ferraz, Michael Lasnik, Alfred Krumphals, Maria Cecilia Poletti

**Affiliations:** 1 Christian Doppler Laboratory for Design of High-Performance Alloys by Thermomechanical Processing, Kopernikusgasse 24, 8010 Graz, Austria; franz.ferraz@tugraz.at (F.M.B.F.); cecilia.poletti@tugraz.at (M.C.P.); 2 Institute of Materials Science, Joining and Forming, Graz University of Technology, Kopernikusgasse 24, 8010 Graz, Austria; 3 voestalpine BÖHLER Aerospace GmbH & Co KG, Mariazellerstraße 25, 8605 Kapfenberg, Austria; Michael.Lasnik@voestalpine.com (M.L.); Alfred.Krumphals@voestalpine.com (A.K.)

**Keywords:** mesoscale model, dislocation density, hot deformation, microstructure, continuous dynamic recrystallization, titanium alloys

## Abstract

Two different mesoscale models based on dislocation reactions are developed and applied to predict both the flow stress and the microstructure evolution during the hot deformation of titanium alloys. Three distinct populations of dislocations, named mobile, immobile, and wall dislocations, describe the microstructure, together with the crystal misorientation and the densities of boundaries. A simple model consisting of production and recovery terms for the evolution of dislocations is compared with a comprehensive model that describes the reactions between different type of dislocations. Constitutive equations connect the microstructure evolution with the flow stresses. Both models consider the formation of a high angle grain boundary by continuous dynamic recrystallization due to progressive lattice rotation. The wall dislocation density evolution is calculated as a result of the subgrain size and boundary misorientation distribution evolutions. The developed models are applied to two near-β titanium alloys, Ti-5553 and Ti-17, and validated for use in hot compression experiments. The differences in the predictability between the developed models are discussed for the flow stress, dislocation densities and microstructure evolutions. Only the comprehensive model can predict the different reactions and their contributions to the evolution of mobile and immobile dislocation densities. The comprehensive model also allows for correlating the elastic strain rate with the softening and hardening kinetics. Despite those differences, the selection of the model used has a small influence on the overall prediction of the subgrain size and the fraction of high angle grain boundaries.

## 1. Introduction

As a high stacking fault energy (SFE) material, the β-phase in titanium and its alloys undergoes a complex microstructure evolution during hot deformation due to dynamic recovery (DRV) and dynamic recrystallization (DRX) [1,2,3,4,5,6,7,8]. Although there are some controversies [1,2,3,4], it is generally accepted that the β-phase undergoes DRV [5] followed by continuous dynamic recrystallization (cDRX) [8] or geometric dynamic recrystallization (gDRX) [6,7] at large strains. cDRX occurs by progressive lattice rotation [6,7] and was also observed in Ti-64 [5], while gDRX occurs at lower strain rates and lower temperatures [6,7].

From discrete dislocation dynamics [9] to phenomenological approaches [10,11], the hot deformation behavior of metallic alloys has been modeled and predicted. Among the most powerful methods to predict the hot deformation of complex parts, the mesoscale models are of great interest [12]. Kocks [13], Bergström [14], Bergström and Roberts [15], Roberts and Bergström [16] and Mecking and Estrin [17] made the initial attempts to develop dislocation density-based models for metal plasticity. Ghoniem et al. [18] proposed a dislocation-based model to describe the dislocation creep behavior of steels, accounting for the several dislocation density reactions that can occur on a mesoscopic scale. A similar dislocation density reaction approach was applied to describe the hot deformation of a Ti-5553 alloy [19] and a Ti-17 alloy [20]. Simpler dislocation density-based models, mostly based on Kock–Mecking formalism [21], were developed to predict the deformation behavior of other Ti alloys. The deformation of a Ti-6Al-4V over a wide temperature range was achieved in Ref. [22], while constitutive equations applied to room temperature deformation describe the complex deformation mechanism, as described in [23]. Picu and Majorell [24] proposed constitutive equations to predict the influence of the chemical composition on the deformation behavior of a Ti-6Al-4V. The Kocks–Mecking formalism was coupled with dDRX to simulate the flow softening observed in the α+β domain [25,26,27]. The approach was extended in Ref. [28] to include Hall–Petch describing the hot deformation behavior of Ti alloys in both α+β and β domains. Babu et al. [29] extended the modeling approach to include the effects of vacancy evolution and globularization of the α-phase. The damage was coupled to dDRX to predict the tensile properties of a TA15 alloy [30]. Although cDRX or gDRX are observed as restoration mechanisms for high stacking fault energy materials, most existing models still consider discontinuous dynamic recrystallization (dDRX) as the deformation mechanism of titanium alloys [25,26,27,28,29,30,31,32,33,34,35,36,37,38,39,40], and also do not consider the deformation in the α+β domain as a biphase plastic deformation [25,26,27,29,30,31,34,35,37,38,39].

This publication aims to compare and discuss the predictability of the well-established Kock–Mecking formalism [21] with a novel model described in Refs. [19,20] based on the work of Ghoniem et al. [18] where different dislocation reactions can be quantified. Both dislocation rate models are combined with a cDRX model and can predict the evolution of any starting microstructure. Moreover, the plastic deformations of both α and β phases are considered in the α+β domain and a change in flow partitioning is related to the flow softening.

## 2. Materials and Methods

Cylindrical samples of 10 mm diameters and 15 mm lengths were cut perpendicular to the forging direction and parallel to the cogging direction from disks of 128 mm and 256 mm diameters from commercial Ti-5553 and Ti-17, respectively. The cogged Ti-5553 presented globular primary α grains, and β grains elongated in the cogging direction with a size ~1 mm in length and ~150 µm in width. The as-received microstructure of the Ti-17 consisted of a lamellar microstructure and prior globular-like β-grains of ~500 µm in length. The β-transus temperature is ~860 °C for Ti-5553 in agreement with the literature [41,42] and ~865 °C for Ti-17. These temperatures were estimated using JMatPro^®^ v.10 for the chemical compositions shown in Table 1.

Hot compression tests were performed using a Gleeble^®^ 3800. Experiments were performed in the α+β phase field at 800 °C, 820 °C and 840 °C for the Ti-5553 and at 810 °C, 830 °C, and 850 °C for the Ti-17. Hot compression of the single β-phase was performed at 880 °C, 900 °C and 920 °C for the Ti-5553, and at 930 °C, 950 °C and 970 °C for the Ti-17. Five different constant true strain rates from 0.001 s^−1^ to 10 s^−1^ were tested. The specimens were heated at 5 °Cs^−1^ up to the testing temperature and held for 15 min before deformation, except for the hot compression at the single β-phase-field for Ti-5553, where the holding time was 5 min. The temperature was measured with a thermocouple type K welded at the surface of the sample, and Ar was used as a protective atmosphere to prevent oxidation and α-case formation. Graphite and tantalum foils were used to minimize the friction of the samples with the anvils. The samples were in situ water-quenched immediately after hot deformation to preserve the deformed microstructure. Some samples were water-quenched immediately after annealing to investigate the microstructure before deformation. The stress-strain data were corrected with respect to adiabatic heating according to the procedure described elsewhere [43,44]. The material density was considered as 4300 kg·m^3^ [45], the specific heat ($C_{p}$ in (J·K^−1^·kg^−1^)) and material conductivity ($K_{w}$ in (W·m^−1^·K^−1^)) of the workpiece are given in Equations (1) and (2), respectively. A thermal conductivity of 10 (W·m^−1^·K^−1^) [44] was considered for a SiN die.

(1) $$C_{p}\;=\;5.82\cdot 10^{-9}\cdot T^{2}-1.12\cdot 10^{-6}\cdot T+6.18\cdot 10^{-3},$$

(2) $$K_{w}\;=\;-0.0109\cdot T+34.22,$$

The samples annealed before deformation and hot compression were cut in half perpendicular to the radial direction, cold mounted, ground using SiC paper, polished using silica oxide polishing suspension (OPS) solution, and vibro-polished before the microstructure analysis. Preparation using a cross-section polisher JEOL SM-09010 (JEOL, Tokyo, Japan) was used for EBSD investigations carried out in Ti-17. A TESCAN Mira3 (TESCAN, Brno, Czech Republic) and a JEOL JSM7001F (JEOL, Tokyo, Japan) electron scanning microscope were used for microstructure investigation. A TSL-OIM Data Collector software package was used for performing the EBSD measurements. The measurements of (500 µm)^2^ with a step size of 0.5 µm to investigate the β-phase were carried out at an acceleration voltage of 30 kV and a spot size of 80 nm using a TESCAN Mira3 (TESCAN, Brno, Czech Republic) microscope. An acceleration voltage of 15 kV and a spot size of 5 nm was used for the measurements of the α and β phases in Ti-17 using a JEOL JSM7001F (JEOL, Tokyo, Japan) microscope. The OIM Analysis v.8 software was used to treat the data. The minimum grain size of 2 µm was chosen to guarantee that each grain consists of at least four measured pixels. Misorientation angles larger than 12° were assigned to high angle grain boundaries (HAGBs). The confidence index was standardized for each grain. The EBSD data were then cleaned, considering a minimum confidence index of 0.5 correlated to the neighbor grains. A maximum of ~5% of the data were cleaned.

## 3. Modeling Strategy

The developed model consists of four main elements: microstructure concept, constitutive equations, plastic strain rate partitioning, and rate equations. The model parameters values and expressions are given in Appendix A.

### 3.1. Microstructure Model

The microstructure was modeled based on its mean properties, i.e., an average subgrain and grain sizes, as well as three different populations of dislocations. The representative microstructure entity named subgrain is considered to be surrounded by low angle grain boundaries (LAGBs) and HAGBs, named subgrain. The LAGBs are considered to be sharp and consisted of *n* sets of dislocations forming a wall [46]. Two populations of dislocations are distinguished within the subgrain: the mobile and the immobile dislocations. All dislocation densities are related to the whole volume of material. The mobile dislocations slide along the active slip systems [47] and accommodate the plastic deformation. Obstacles, such as other dislocations, boundaries, or phase interfaces [48], block the immobile dislocations. They do not contribute directly to plastic deformation and are responsible for the hardening [49].

The misorientation distribution and the volumetric boundary density define, respectively, the fractions and size of the subgrain. In the case of a substantial fraction of LAGB, a subgrain is surrounded mainly by LAGB. In an ideally fully recrystallized material, the fraction of LAGB is negligible, and the subgrain size is coincident with the grain size. The full description of the microstructure is achieved by including the misorientation distribution. This is modeled as a sum of Rayleigh and Mackenzie [50] distributions [19]. The Rayleigh distribution ($\Theta_{R}\left(\theta \right)$) accounts for the misorientation caused by the LAGB formed during deformation. The increase in boundary misorientation leads to the evolution of HAGB. The Mackenzie distribution ($\Theta_{M}\left(\theta \right)$) considers a fully recrystallized material with random texture. Texture evolution is not considered in the developed models. The initialization of the microstructure is explained in Appendix B, and the microstructural features values for the Ti-5553 and Ti-17 are defined in Table A1 in Appendix A.

### 3.2. Constitutive Equations

Constitutive equations are required to couple the microstructure with the flow stress (σ). The thermal, $\sigma_{th}$, and athermal, $\sigma_{ath}$, stresses are considered as the constituents of the flow stress for each phase, Equation (3). Any effect of dislocation pile-up along the grain boundary (Hall–Petch effect) is considered negligible due to the small Hall–Petch coefficient of typical Ti alloys at high temperatures [28,51]. However, this effect must be considered for deformation at lower temperatures.

(3) $$\sigma_{x}\;=\sigma_{ath_{x}}+\sigma_{th_{x}},\;\;\;\;x\;=\alpha ,\;\beta$$

The athermal stress is expressed according to Equation (4) as a function of the immobile, mobile, and wall dislocation densities ($\rho_{i}$, $\rho_{m}$, and $\rho_{w}$, respectively), the Taylor constant (*α*), the Taylor factor (*M*), and the shear modulus (*G*).

(4) $$\sigma_{ath_{x}}\;=\;\alpha MGb\sqrt{\rho_{i_{x}}+\rho_{m_{x}}+F_{w_{x}}\rho_{w_{x}}},\;\;\;\;x\;=\alpha ,\;\beta$$

The Taylor constant is considered as 0.1 for both alloys, and its low value is attributed to the lattice resistance due to the interaction between parallel dislocations as the main barrier for dislocation movement at high temperature [52]. $F_{w}$ is described in Appendix C. The thermal stress is calculated using the fitted yield stress ($\sigma_{YS}$), Equation (5). The procedure to obtain $\sigma_{YS}$ is described in Appendix D.

(5) $$\sigma_{th_{x}}\;=\;\sigma_{YS_{x}}-\sigma_{ath_{x}}^{0},\;\;\;\;x\;=\alpha ,\;\beta$$

$\sigma_{ath}^{0}$ is calculated considering initial dislocation densities. The $\sigma_{th}$ is constant for a constant temperature and strain rate since the $\sigma_{YS}$ does not vary with the temperature and the strain rate.

### 3.3. Load Partitioning

The deformation rate of hard platelets of the α-phase is different than the deformation rate of the soft β-matrix. As explained in [53,54,55], iso-strain, iso-stress, and/or iso-power load conditions have to be considered. The change in load transfer between both phases explains the observed flow softening in the α+β domain. The Ti-5553 and the Ti-17 exhibit an initial globular and lamellar structure, respectively. Thus, it is proposed:

- Ti-5553—the rotation and accommodation of plastic deformation in the α-particles up to a steady-state condition lead to the change from iso-power to iso-stress regimes;
- Ti-17—the process of dynamic α-globularization leads to a change from iso-strain to iso-stress regimes.

The eventual formation of texture is not considered in the model. The change in flow partition mechanisms is here used to describe the combined effects that lead to flow softening during deformation in the α+β domain. The equations and assumptions of iso-strain, iso-stress, and/or iso-power load conditions are explained in Appendix E.

#### 3.3.1. Overall Stress

The overall stress is calculated using a rule of mixtures, Equation (6) as a function of the fraction of α-phase $F_{v}$, and the stress in the α ($\sigma_{\alpha }$) and β ($\sigma_{\beta }$) phases.

(6) $$\sigma \;=\;F_{v}\sigma_{\alpha }+\;\left(1-F_{v}\right)\sigma_{\beta }$$

#### 3.3.2. Fraction of Material in Iso-Stress Regime

In the case of the Ti-5553, the volumetric fraction of α and β phases ($f_{iso-\sigma }$) that evolves from iso-power to iso-stress load partitioning is given empirically by Equation (7) as a function of the overall strain (*ε*) and strain rate ($\dot{\varepsilon }$).

(7) $$f_{iso-\sigma }\;=\;1-\exp\left(-A_{iso-\sigma }{\left(\frac{\dot{\varepsilon }}{{\dot{\varepsilon }}_{ref}}\right)}^{n_{s}}\varepsilon^{n_{s}}\right)$$

$A_{iso-\sigma }$ and $n_{s}$ are fitting parameters and ${\dot{\varepsilon }}_{ref}$ is a reference strain rate.

In the case of the Ti-17, the $f_{iso-\sigma }$ is the fraction of α-globularization ($f_{glob}$). Finally, the overall strain rate for each phase (${\dot{\varepsilon }}_{x}$) is calculated as a rule of mixtures of both contributions, Equation (8).

(8) $${\dot{\varepsilon }}_{x}=\left(1-f_{iso-\sigma }\right){\dot{\varepsilon }}_{x}^{y}+f_{iso-\sigma }{\dot{\varepsilon }}_{x}^{\sigma },\;\;\;\;x=\alpha ,\;\beta ;\;y=W\;(\mathrm{Ti}-5553)\;\mathrm{or}\;\varepsilon \;(\mathrm{Ti}-17)$$

### 3.4. Dynamic α-Globularization

The dynamic α-globularization consists of the formation of boundaries within the α-platelets and the movement of α/β interfaces leading to the formation of globular α-particles [56,57]. It is proposed that initially the load is transferred to the α-platelets following the iso-strain regime. With the onset of the α-globularization, the strain rate in the α-phase reduces, reaching the iso-stress regime at the flow steady-state condition.

An α-platelet of mean aspect ratio ($A_{R}$) with a mean thickness ($t_{\alpha }$) and mean width ($w_{\alpha }\;=\;t_{\alpha }\cdot A_{R}$) is considered. The dynamic α-globularization consists of the formation of boundaries within the α-platelet due to plastic deformation followed by the formation of new α/β interfaces by the migration of grooved interfaces with a velocity $v_{glob}$. Thickening of the α-particles can occur, leading particles with an aspect ratio of one. The overall boundary density within the α-phase is a sum of the production of new boundaries modeled similarly to cDRX [46], minus the consumption of existing boundaries within the α-phase due to α/β interface movement through the HAGBs within the α-phase, Equation (9).

(9) $$\frac{dS_{v_{\alpha }}\;}{dt}\;=\;\alpha_{CDRX}\frac{b}{n\theta_{0}}\Delta \rho_{cDRX}{\dot{\varepsilon }}_{p_{\alpha }}-\frac{f_{BG}f_{HAGB_{\alpha }}S_{v_{\alpha }}v_{glob}}{t_{\alpha }}$$

${\dot{\varepsilon }}_{p_{\alpha }}$ is the plastic strain rate in the α-phase, $f_{BG}$ is the globularized fraction, $S_{v_{\alpha }}$ is the boundary surface density within the α-platelet, $S_{glob}$ is of the α-phase, and $v_{glob}$ is the velocity for α/β interface movement. Equation (10) describes the rate of the production of α/β interface density formed during dynamic globularization ($dS_{glob}/dt$).

(10) $$\begin{array}{cc} \frac{dS_{glob}\;}{dt}\;=\;\frac{f_{BG}f_{HAGB_{\alpha }}S_{v_{\alpha }}v_{glob}}{t_{\alpha }}\;| & S_{glob}\;=\;\int_{0}^{t}\left(\frac{dS_{glob}\;}{dt}\right)dt \end{array}$$

Equation (11) describes the total boundary density formed within the α-platelet.

(11) $$S_{TOTAL_{\alpha }}\;=\;S_{v_{\alpha }}+S_{glob}$$

Full globularization of an α-platelet occurs when $S_{v_{\alpha }}$ = 0. Equation (12) gives the globularization fraction.

(12) $$f_{glob}\;=\;\frac{S_{glob}}{S_{TOTAL_{\alpha }}}$$

Appendix F describes the dynamic α-globularization in more detail.

### 3.5. Rate Equations and Microstructure Evolution

The evolution of the internal variables is expressed as differential equations and is solved using numerical incremental steps for both α and β phases. In this manuscript, two models to describe the rate equations are compared: (1) a more general and simple model based on the work of Kocks and Mecking [21] (herein named “Model KM”); (2) a model with descriptions of dislocation reactions based on the work of Ghoniem et al. [18] (herein named “Model G”).

#### 3.5.1. Mobile Dislocation Density

Model KM

The mobile dislocation density is considered to be constant at a constant strain rate, in agreement with the Orowan’s relationship (Equation (13)). The experimental strain rate ($\dot{\varepsilon }$) and the plastic strain rate (${\dot{\varepsilon }}_{p}$) are the same.

(13) $$v_{g_{x}}\;=\;\frac{M_{x}{\dot{\varepsilon }}_{p_{x}}\;}{b_{x}\rho_{m_{x}}},\;\;\;\;x\;=\alpha ,\;\beta$$

The mobile dislocation density is assumed invariable with respect to plastic strain rate and strain because:

- the developed model does not consider any effect of texture and change in the Taylor factor (*M*);
- the Burgers vector (*b*) is constant during deformation;
- the glide velocity is independent of strain if the plastic strain rate and temperature are kept constant.

Thus, the ratio of the glide velocities for two different strain rates equals the ratio of the strain rates.

Model G

Equation (14) describes the rate for mobile dislocation density. The terms were initially proposed by [18]. The terms related to the production of dislocations at subgrain boundaries and the movement of HAGBs during cDRX are further incorporated into the model as described in Ref. [19].

(14) $$\begin{array}{c} \frac{d\rho_{m_{x}}}{dt}\;=\;\rho_{m_{x}}^{3/2}v_{g}+\frac{\delta_{SG_{x}}\rho_{m_{x}}\Phi_{sg_{x}}v_{g_{x}}}{\lambda_{m_{x}}^{2}}-\frac{\rho_{m_{x}}v_{g_{x}}}{\Phi_{sg_{x}}}-8\frac{\rho_{m_{x}}v_{c}{}_{m_{x}}}{\lambda_{m_{x}}}-\delta_{DRV_{x}}\rho_{m_{x}}\left(\rho_{m_{x}}+\rho_{i_{x}}\right)v_{g_{x}}\\ -\rho_{m_{x}}S_{v_{x}}f_{HAGB_{x}}v_{HAGB_{x}},\;\;\;\;x\;=\alpha ,\;\beta \end{array}$$

The production of mobile dislocations due to Read’s source and at subgrain boundaries corresponds to the first and second terms, respectively. The third, fourth and fifth terms correspond to the immobilization of mobile dislocations at subgrain boundaries, the static recovery due to climb, and the dynamic recovery, respectively. The last term is related to the consumption of mobile dislocations due to the movement of HAGBs during cDRX. $v_{g}$ is the glide velocity, $\lambda_{m}$ and $\lambda_{i}$ are the inter-dislocation distance for the mobile and immobile dislocations, respectively, $v_{c}{}_{m}$ and $v_{c}{}_{i}$ are the climb velocities for the mobile and immobile dislocations, respectively, and $\delta_{DRV}$ is the critical distance of dislocation annihilation via DRV. If the production of mobile dislocations at subgrain boundaries is neglected, the immobilization at subgrain boundaries can lead to the annihilation of all mobile dislocation. For simplification, $\delta_{SG}$ is an internal variable adjusted at each step to maintain a constant glide velocity during plastic deformation at a given strain rate and temperature. $f_{HAGB}$ is the fraction of HAGBs ($f_{HAGB}\;=\;1-f_{LAGB}$). The velocity of HAGBs ($v_{HAGB}$) is described in Appendix G. The ${\dot{\varepsilon }}_{p}$ is determined as a function of the mobile dislocation density by the Orowan’s relationship (Equation (13)), where ${\dot{\varepsilon }}_{p}$ is the output. Here, the difference with respect to the “Model KM” is that the glide velocity is calculated phenomenologically according to Equation (15). The expression describes the mechanisms of strengthening [18] as a function of the activation energy for gliding ($W_{g}$), the thermal stress, the atomic volume (Ω), the Boltzman’s contant ($k_{B}$) and the temperature (T). The pre-factor ($a_{1}$) is calculated as described in Ref. [19].

(15) $$v_{g_{x}}\;=\;a_{1_{x}}\exp\left(-\frac{W_{g_{x}}}{k_{B}T}\right)\frac{\sigma_{th_{x}}\Omega }{k_{B}T},\;\;\;\;x\;=\alpha ,\;\beta$$

The experimental strain rate equals the sum of the plastic strain rate and the elastic strain rate (${\dot{\varepsilon }}_{e}$). Consequently, the variation in the flow stress at each iteration is expressed according to the derivative of the Hook’s law, Equation (16), as a function of the Young modulus (*E*).

(16) $${\dot{\sigma }}_{x}\;=\;E_{x}\left({\dot{\varepsilon }}_{x}-{\dot{\varepsilon }}_{p_{x}}\right),\;\;\;\;x\;=\alpha ,\;\beta$$

#### 3.5.2. Immobile Dislocation Density

Model KM

Equation (17) describes the immobile dislocation density rate using a modified expression from Kocks and Mecking [21] and Gourdet and Montheillet [46].

(17) $$\frac{d\rho_{i_{x}}}{dt}\;=\;h_{1_{x}}\;{\dot{\varepsilon }}_{p_{x}}-h_{2_{x}}\;\rho_{i_{x}}\;{\dot{\varepsilon }}_{p_{x}}-\rho_{i_{x}}S_{v_{x}}f_{HAGB_{x}}v_{HAGB_{x}},\;\;\;\;x\;=\alpha ,\;\beta$$

The rate equation has the following terms:

- a term with a hardening coefficient (*h_1_*) to describe the immobilization of mobile dislocations at forest dislocations, cross-slip around obstacles [47], and the formation of diploes of mobile dislocations with an anti-parallel Burgers vector [49];
- a term with a recovery coefficient (*h_2_*) to describe the consumption of immobile dislocations that occurs due to their rearrangement and annihilation [40] by DRV;
- a softening term to describe the consumption of immobile dislocations that are swept by the movement of HAGBs during cDRX.

The definitions of the terms *h*_1_ and *h*_2_ are given in Appendix H.

Model G

The rate equations for immobile dislocation are modified in Ref. [19] from Ref. [18] to include the consumption of immobile dislocations due to the movement of HAGBs during cDRX; Equation (18).

(18) $$\frac{d\rho_{i_{x}}}{dt}\;=\;\frac{\rho_{m_{x}}v_{g_{x}}}{\Phi_{sg_{x}}}-8\frac{\rho_{i_{x}}v_{c}{}_{i_{x}}}{\lambda_{i_{x}}}-\delta_{DRV_{x}}\rho_{m_{x}}\rho_{i_{x}}-\rho_{i_{x}}S_{v_{x}}f_{HAGB_{x}}v_{HAGB_{x}},\;\;\;\;x\;=\alpha ,\;\beta$$

The production of immobile dislocations occurs via the immobilization of mobile dislocations (first term), while SRV and DRV are responsible for the annihilation of immobile dislocations (second and third terms, respectively).

#### 3.5.3. Boundary Density and Misorientation Distribution

The model of cDRX [46] assumes that an amount of dislocations ($\Delta \rho_{cDRX}$) either are rearranged into LAGBs or migrate into existing LAGBs, increasing their misorientation. The boundary density evolution is given by Equation (19) [46]. $\alpha_{CDRX}$ is the fraction of dislocations that form new LAGBs, while (1 − $\alpha_{CDRX}$) is the fraction that increases the misorientation of existing LAGBs. $\theta_{new}$ is the mean misorientation angle of a newly formed boundary. The second term corresponds to the boundary surface density that is swept by the movement of HAGBs during cDRX.

(19) $$\frac{dS_{v_{x}}}{dt}=\alpha_{CDRX_{x}}\frac{b_{x}}{n_{x}\theta_{new_{x}}}\Delta \rho_{cDRX_{x}}\;-\;S_{v_{x}}{}^{2}f_{HAGB_{x}}v_{HAGB_{x}},\;\;x=\alpha ,\;\beta$$

The evolution of the misorientation distribution is given by the increase in the mean boundary misorientation angle of the Rayleigh distribution, and is given in Equation (20) [46].

(20) $$\frac{d\bar{\theta_{R_{x}}}}{dt}\;=\;\left(1-\alpha_{CDRX}{}_{x}\right)\frac{b}{n_{x}S_{v_{x}}}\Delta \rho_{cDRX_{x}},\;\;x=\alpha ,\;\beta$$

The increase in the average boundary misorientation of the Rayleigh distribution leads to a change in $f_{R}$ (Equation (A12) in Appendix B). The boundary misorientation angle distribution is calculated using Equation (A13) for the new values of $f_{R}$ and $A_{1}$.

Model KM

The amount of dislocations that contribute to the boundary density and misorientation distribution evolution is the amount of recovered immobile dislocations, Equation (21).

(21) $$\Delta \rho_{cDRX}\;=\;h_{2}\;\rho_{i}\;{\dot{\varepsilon }}_{p}\Delta t$$

Model G

The amount of dislocations that contribute to the boundary density and misorientation distribution evolution is a fraction ($f_{CDRX}$, see Appendix A) of the mobile and immobile dislocations that are annihilated by SRV and DRV, and is given in Equation (22).

(22) $$\Delta \rho_{cDRX}=f_{CDRX}\left(\overset{SRV}{\overbrace{8\frac{\rho_{m}v_{c}{}_{m}}{\lambda_{m}}}}+\overset{DRV}{\overbrace{\delta_{DRV}\rho_{m}\left(\rho_{m}+\rho_{i}\right)v_{g}}}+\overset{SRV}{\overbrace{8\frac{\rho_{i}v_{c}{}_{i}}{\lambda_{i}}}}+\overset{DRV}{\overbrace{\delta_{DRV}\rho_{m}\rho_{i}v_{g}}}\right)\Delta t$$

#### 3.5.4. Wall Dislocation Density, Grain and Subgrain Size

The evolution of the boundary dislocations density is related to the evolution of the subgrain size; Equation (9). The grain size ($G_{s}$) and the subgrain size ($\Phi_{sg}$) of the β-phase are calculated according to Equations (23) and (24), respectively.

(23) $$G_{s}\;=\;\frac{2f_{HAGB}}{S_{v}}$$

(24) $$\Phi_{sg}\;=\;\frac{2}{S_{v}}$$

Finally, the wall dislocation density ($\rho_{w}$) is updated according to Equation (A14) at each iteration for both phases.

## 4. Results and Discussion

The measured and simulated flow stresses are compared for both Ti-5553 and Ti-17 alloys. Dynamic α-globularization and cDRX are interpreted from the experimental results. The differences in the predictions of the two developed models are discussed and compared.

### 4.1. Measured Microstructure

DRV and SRV occur by the annihilation and reorganization of dislocations during plastic deformation. The formed boundaries:

- in the platelets of the α-phase promote α/β interface migration and the progressive globularization of the α-phase;
- within the β-phase as the consequence of the progressive increase in boundary misorientation angle.

**Figure 1:**
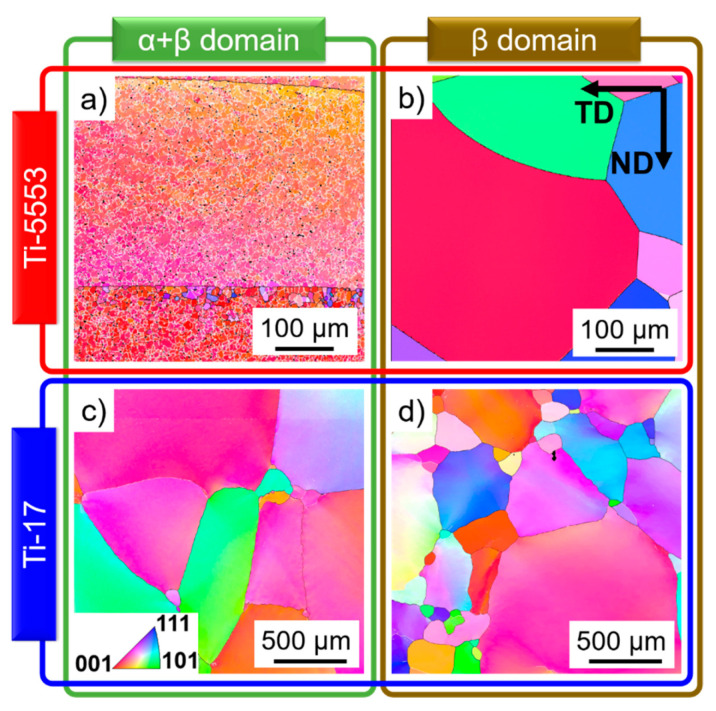
Microstructures before deformation of the (**a**,**b**) Ti-5553 and (**c**,**d**) Ti-17 after heat treatment at (**a**) 820 °C for 15 min, (**b**) 920 °C for 5 min, (**c**) 810 °C for 15 min, and (**d**) 970 °C for 15 min. Only the β-phase is indexed in the electron backscattered difraction (EBSD) inverse pole figure maps, and the black lines are HAGBs.

**Figure 2:**
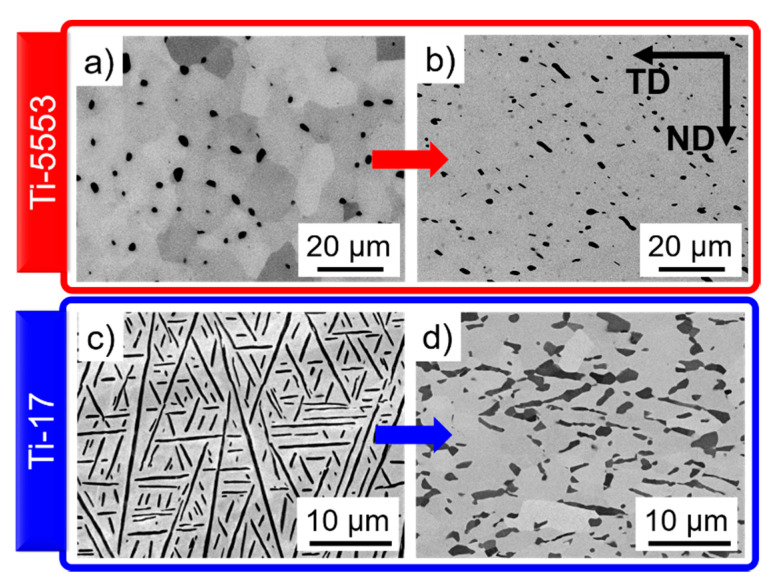
Microstructures of the (**a**,**b**) Ti-5553 and (**c**,**d**) Ti-17. Microstructures after heat treatment for 15 min at (**a**) 820 °C, (**c**) 810 °C. Microstructures after deformation at 0.001 s^−1^ and (**b**) 820 °C, (**d**) 810 °C. The α- and β-phases are the darker and lighter phases, respectively.

**Figure 3:**
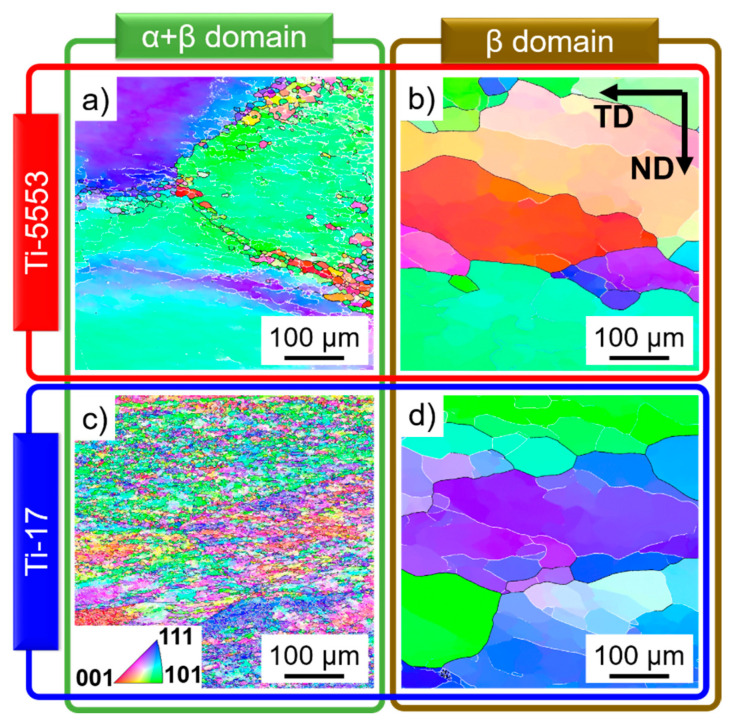
Microstructures of the (**a**,**b**) Ti-5553 and (**c**,**d**) Ti-17 after deformation at 0.001 s^−1^ and (**a**) 820 °C, (**b**) 920 °C, (**c**) 810 °C, (**d**) 970 °C. Only the β-phase is indexed in the EBSD inverse pole figure maps, and the black lines are HAGBs.

Figure 1, Figure 2 and Figure 3 show typical microstructures before and after deformation for the two near-β alloys. The microstructures of the α- and β-phases evolve as follows.

#### 4.1.1. α-Phase

- Ti-5553 exhibits an initial globular α-phase microstructure (Figure 2a), and slightly elongated α-particles due to plastic deformation after hot deformation at 820 °C and 0.001 s^−1^; Figure 2b. Ti-17 has an initial lamellar α-phase; Figure 2c. The α-phase becomes partially dynamically globularized after hot deformation at 810 °C and 0.001 s^−1^; Figure 2d.

#### 4.1.2. β-Phase

- Ti-5553: Figure 1a shows elongated β-grains perpendicular to the forging direction before deformation at 820 °C. LAGBs and HAGBs were formed in the β-phase after deformation at 820 °C and 0.001 s^−1^; Figure 3a. The dissolution of the α-phase beyond 920 °C and the higher HAGBs mobility promotes the formation of a fully recrystallized microstructure after annealing, Figure 1b;
- Ti-17: Figure 1c,d show partially statically recrystallized β-grains before deformation at 820 °C and 970 °C, respectively. Figure 3c shows a well-established β-subgrain structure with newly formed HAGBs between the partially globularized α-particles after deformation at 810 °C and 0.001 s^−1^.
- Figure 3b,d show larger recovered subgrains with very few newly formed HAGBs for the Ti-5553 and Ti-17 deformed in the β-domain.

### 4.2. Identification of the Restoration Phenomena

**Figure 4:**
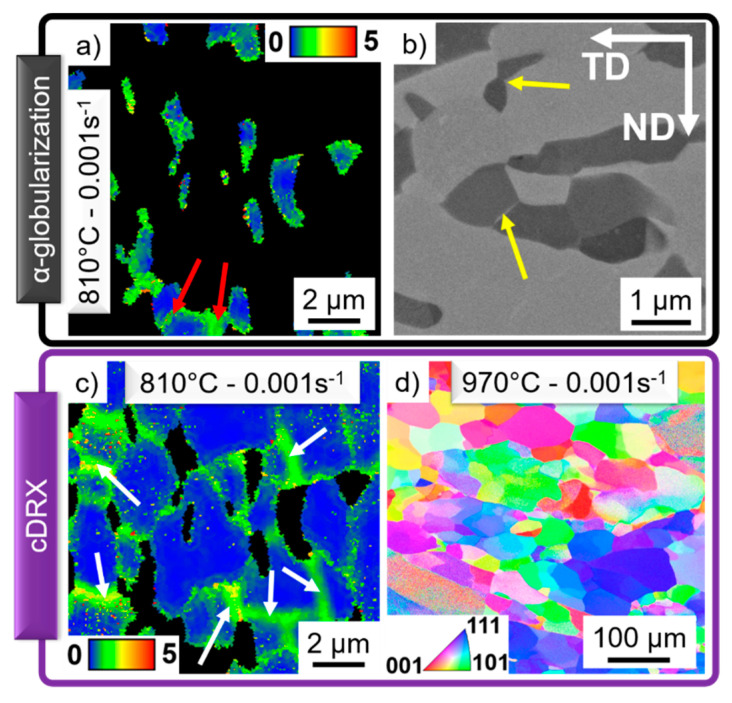
Typical microstructures of the Ti-17 deformed at 0.001 s^−1^ and (**a**–**c**) 810 °C, (**d**) 970 °C; representing the process of (**a**,**b**) dynamic α-globularization; (**c**,**d**) cDRX.

The intricate α-lamellar structure in the Ti-17 leads to the rotation, kinking or bending of the lamellas during plastic deformation. Dislocations are formed and rearranged into LAGBs as indicated in the KAM map in Figure 4a by the red arrows. If boundaries are formed within the α-phase, migration of the α/β interface occurs, leading to the formation of globular α-grains, as shown in Figure 4b (yellow arrows).

The high SFE of the β-phase facilitates the reorganization of dislocations into subgrain boundaries via DRV. The microstructure is comparable for the Ti-5553 deformed at 920 °C (Figure 3b) and the Ti-17 deformed at 970 °C (Figure 3d). The low fraction of newly formed HAGBs indicates the early stage of cDRX for those deformation conditions. On the other hand, the behavior of the β-phase deformed in the α+β domain notably differs between the Ti-5553 (Figure 3a) and the Ti-17 (Figure 3d). Figure 4 shows that the high values of KAM are only associated with LAGBs (white arrows) and not accumulated at α/β interfaces. Thus, the rotation, bending or kinking of the α-phase not only leads to α-globularization but also promotes the formation of boundaries within the β-phase [20,57]. The globular and lower fraction of the α-phase leads to an earlier stage of cDRX of the β-phase for the Ti-5553 (Figure 3a) in comparison to the larger α-fraction with lamellar morphology in Ti-17 (Figure 3c). Figure 4d shows a grain reference orientation deviation axis map. The different axis of rotation of each subgrain shows that progressive lattice rotation leads to an increment in boundary misorientation, i.e., cDRX.

### 4.3. Flow Curves

**Figure 5:**
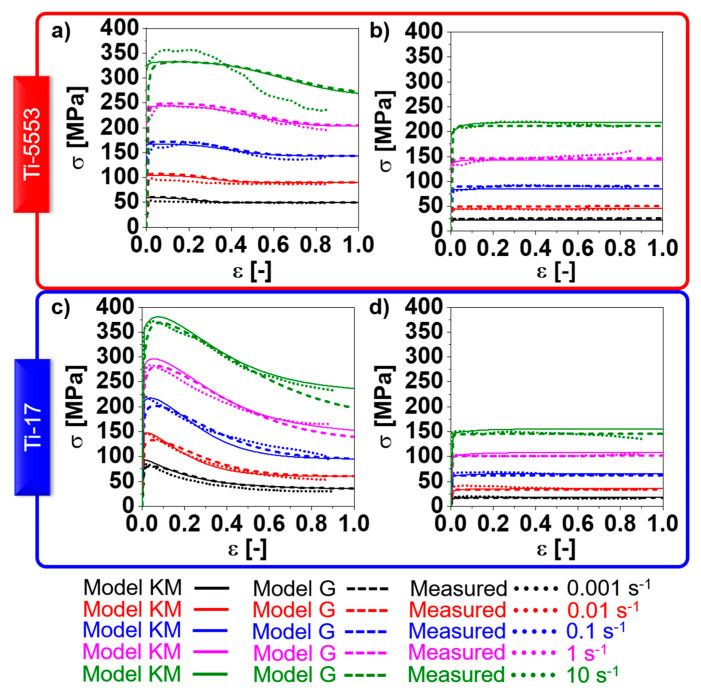
Simulated flow curves using the “Model KM” and the “Model G” compared to the measured ones for (**a**,**b**) Ti-5553 and (**c**,**d**) Ti-17 at (**a**) 800 °C, (**b**) 920 °C, (**c**) 810 °C, and (**d**) 970 °C.

Figure 5 shows the flow curves for Ti-5553 (Figure 5a,b) and Ti-17 (Figure 5c,d). The initial load partitioning regime of the initial α-globular Ti-5553 microstructure corresponds to the iso-power regime, while the lamellar Ti-17 is associated with an initial iso-strain load partitioning. The steady-state load partition is the iso-stress regime. The influence of the hard α-phase in the overall flow stress is stronger for the iso-strain regime compared to the iso-power one. Thus, Ti-17 exhibits a more pronounced hardening as well as softening in the α+β-domain compared to the Ti-5553. For the β-domain, both alloys exhibited a fast work-hardening reaching a plateau-like steady-state.

**Figure 6:**
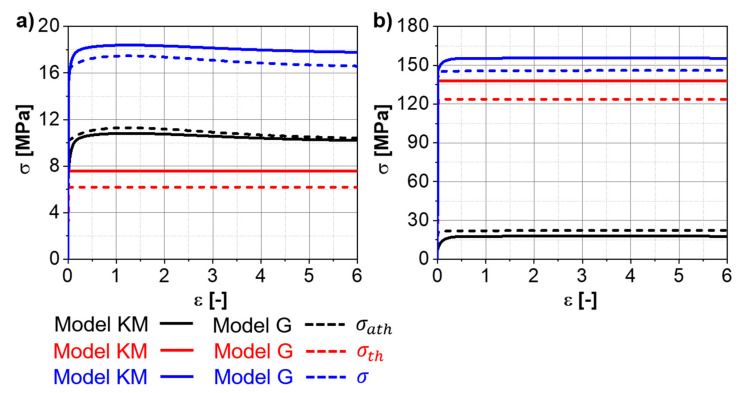
Simulated stress components ($\sigma_{ath}$ and $\sigma_{th}$ ) and total stress (σ ) for the Ti-17 deformed at 970 °C and (**a**) 0.001 s^−1^, (**b**) 10 s^−1^ up to large strains.

The overall differences in the flow stress evolution between the “Model KM” and the “Model G” are minor. Figure 6a,b shows the evolution of the stress components predicted for the Ti-17 deformed at 970 °C, and 0.001 s^−1^ and 10 s^−1^, respectively, up to large strains. A slight softening is observed at 0.001 s^−1^ and corresponds to the decrement of the factor of strengthening ($F_{w}$, see Appendix C) due to the progressive increase in the average boundary misorientation angle. The differences between “Model KM” and “Model G” for the thermal stress are related to the different initial dislocation densities assumed as initial values for each model. However, if both models are initialized with the same dislocation densities, there is no difference in thermal stress between “Model KM” and “Model G”. “Model G” exhibits a faster increase in the athermal stress at the beginning of deformation compared to “Model KM”.

**Figure 7:**
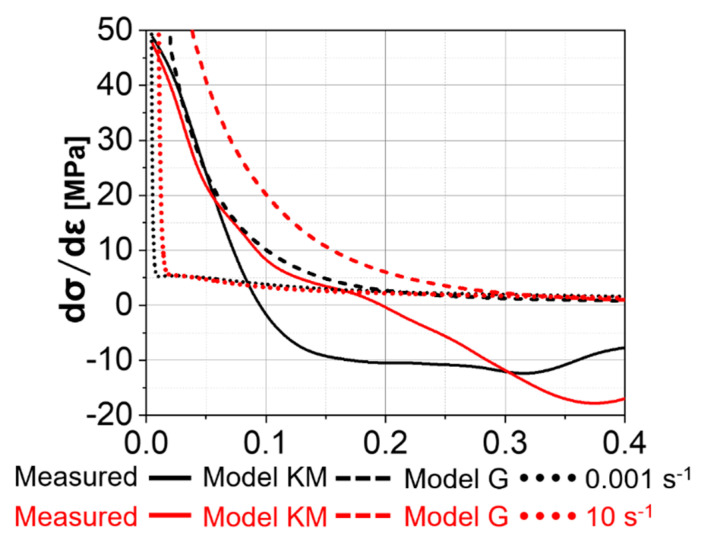
Derivative of the total stress with respect to the strain for the Ti-17 deformed at 970 °C with the strain rates of 0.001 s^−1^ and 10 s^−1^ and for ”Model KM” and “Model G”.

The derivative of the flow stress with respect to the strain is shown in Figure 7 for the Ti-17 deformed at 970 °C. “Model G” predicts a notably faster work-hardening at the beginning of deformation compared to “Model KM” for a given strain rate. The measured values are closer to “Model KM” than to “Model G”. The lower the strain rate, the smaller the strain required to achieve the same value of the derivative at the beginning of the deformation.

### 4.4. Microstructure Evolution Predictions

“Model G” can predict dislocation reactions. Moreover, the existing fitting parameters $\delta_{SG}$ and $\delta_{DRV}$ can be associated with a physical concept. The $\delta_{SG}$ is the fraction of the mobile dislocations that contribute to the production of new mobile dislocations at boundaries, and $\delta_{DRV}$ is a critical distance for the annihilation of dislocations via DRV, thus is not smaller than the Burgers vector, but is in the µm or nm range. “Model G” can predict the evolution of internal variables, such as climb velocity, that are not included in “Model KM” due to simplified dislocation evolution rates. Moreover, every term of the dislocation rate represents a different phenomenon, of which the contribution can be identified and correlated to the deformation conditions (temperature, strain and strain rate). The following chapters discuss the differences between both models in terms of the prediction of the evolution of the α- and β-phases.

#### 4.4.1. α-Phase

**Figure 8:**
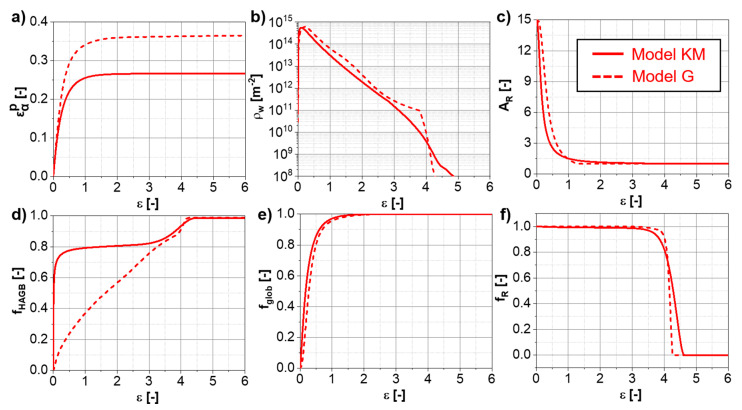
Evolutions of the α-phase for the Ti-17 deformed at 810 °C and 0.001 s^−1^ predicted up to large strains using “Model KM” and “Model G”: (**a**) plastic deformation, (**b**) wall dislocation density, (**c**) α-aspect ratio, (**d**) fraction of HAGB, (**e**) α-globularized fraction, (**f**) fraction of Rayleigh distribution.

Figure 8 shows the influence of the different models on the predictions of the α-evolution for Ti-17 deformed at 810 °C. A fast increase in the strain in the α-phase occurs at the beginning of deformation (Figure 8a) during the iso-strain regime. With the increase in the fraction of globularization (Figure 8e), the strain rate in the α-phase decreases drastically and the iso-stress regime becomes active. The globularization fraction of 100% does not necessarily correspond to a steady-state aspect ratio of 1 (Figure 8c) or that the formation of boundaries within the α-phase is finished (Figure 8d). The fraction of Rayleigh boundary misorientation distribution within the α-phase (Figure 8f) requires larger strains to reach negligible values compared to the globularization fraction.

The differences between “Model KM” and “Model G” for the evolution of the microstructure features for the α-phase are relatively large—a faster α-globularization is predicted by “Model KM” because a faster increase in average boundary misorientation is calculated. The differences between both models are associated with the difference in the amount of dislocations that contribute to the cDRX predicted by both models. They are adjusted by the constant $f_{CDRX}$ for “Model G”, while the behavior and kinetics are given by $\alpha_{CDRX}$ and $\theta_{0}$, $A_{glob}$ and $B_{glob}$. The difference in the glide velocity predictions between “Model KM” and “Model G” leads to the difference in the kinetics of the α/β interface movement related to the constant $A_{glob}$. The slight differences in the flows stress evolutions between “Model KM” and the “Model G” in the α+β-domain in Figure 5 are related to the different α-globularization kinetics predicted by each model.

#### 4.4.2. β-Phase

**Figure 9:**
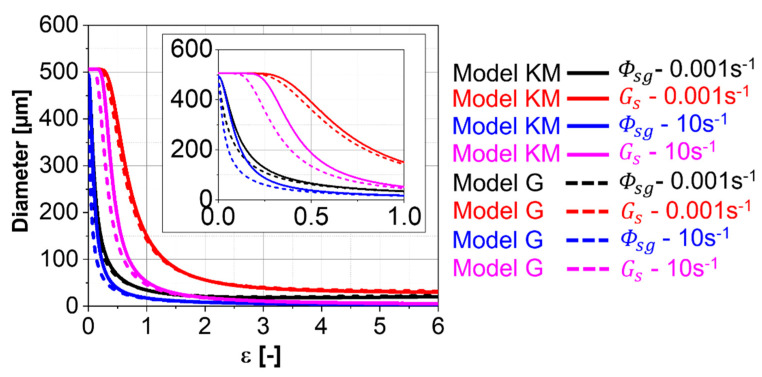
Simulated β-grain and β-subgrain sizes for the Ti-17 deformed at 970 °C predicted using “Model KM” and “Model G”.

Figure 9 shows the grain and subgrain size evolutions for Ti-17 deformed at 970 °C and different strain rates. A fast decrease in subgrain size occurs at the beginning of deformation due to the formation of LAGBs. After a specific strain, the average grain size starts to decrease due to the formation of new HAGBs. The grain size finally decreases with slower kinetics at larger strains, reaching a steady-state subgrain size. The difference in kinetics between the “Model KM” and the “Model G” is associated with the difference in the amounts of dislocations that contribute to the cDRX predicted by both models. They are adjusted by the constant $f_{CDRX}$ for “Model G”, while the behavior and kinetics are given by $\alpha_{CDRX}$ and $\theta_{0}$. However, negligible differences can be observed in the behavior since the same cDRX rate equations are used for both models.

**Figure 10:**
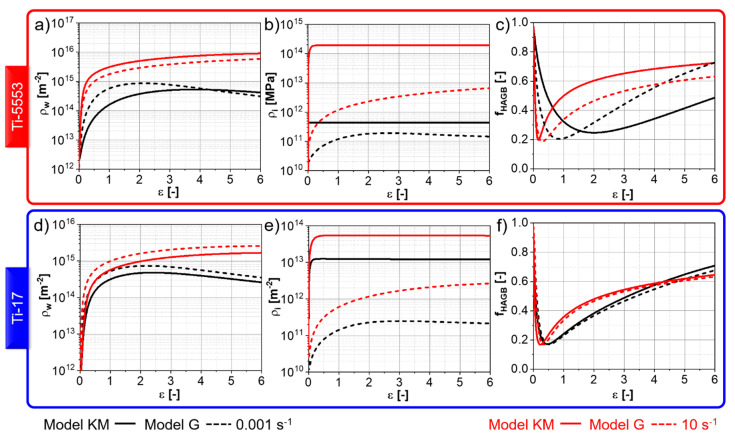
Simulated evolutions of (**a**,**d**) immobile dislocation density; (**b**,**e**) wall dislocation density; (**c**,**f**) fraction of HAGBs for the (**a**–**c**) Ti-5553 deformed at 920 °C and (**d**–**f**) Ti-17 deformed at 970 °C.

Figure 10 shows the evolutions of the immobile and wall dislocation densities, as well as the fraction of HAGBs. Wall dislocations (Figure 10b,e) are rapidly produced due to the reorganization of immobile dislocations in the case of “Model KM” and due to the reorganization of mobile and immobile dislocations via SRV and DRV in case of “Model G”. Higher strain rates lead to faster kinetics initially for the formation of wall dislocation due to the higher rates of DRV. A maximum wall dislocation density follows the rapid increase. It corresponds to the strain whereat the consumption counterbalances the production of new LAGBs. After this maximum, a progressive decrease occurs. Higher strain rates form smaller subgrains sizes (Figure 9). Consequently, a higher rate of dislocation is required to evolve the formed LAGBs. Thus, the higher the strain rate, the slower the pace at which the wall dislocation density reaches the steady state. The fraction of HAGBs shows a fast initial decrease related to the formation of new LAGBs, followed by a valley that corresponds to the strain whereat the boundary misorientation distribution yields the maximum fraction of LAGBs.

The differences are more marked in the evolution of the immobile dislocation density between “Model KM” and “Model G”. “Model KM” predicts a rapid increase in immobile dislocation followed by saturation. Thus, the fast production of immobile dislocations at the early stage of deformation is balanced by their annihilation via DRV. The “Model G” predicts a more complex behavior for the immobile dislocation density evolution. An initial increase occurs due to the immobilization of mobile dislocations at LAGBs and HAGBs. The immobilization competes with the consumption of immobile dislocations via SRV due to the climb, DRV and movement of HAGBs. The immobile dislocation density does not reach an apparent plateau because the subgrain size varies during deformation (Figure 9), and so does also the immobilization rate. It is also notable that the predicted immobile dislocation density using “Model G” is smaller compared to “Model KM”. The main difference is that the Kocks–Mecking formalism [21] adopted in “Model KM” considers the production of immobile dislocations to be independent of the microstructure features. In contrast, in “Model G” the production rate of immobile dislocation density is dependent on the boundary density.

**Figure 11:**
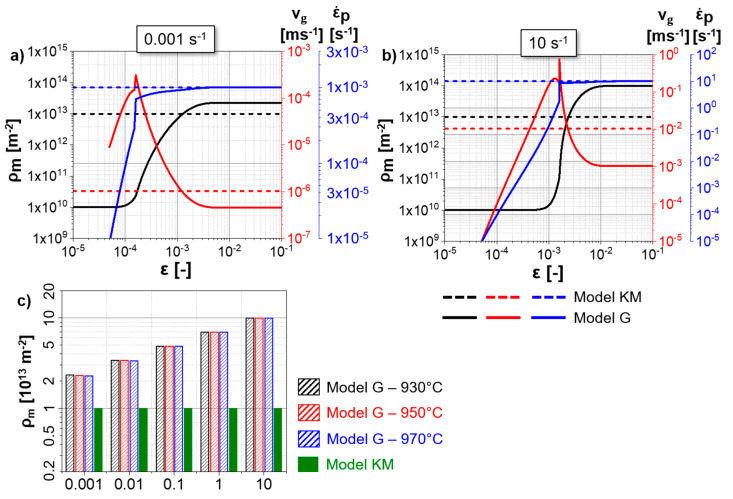
Evolutions of the mobile dislocation density, glide velocity and plastic strain rate predicted by “Model G” for Ti-17 deformed at 970 °C and (**a**) 0.001 s^−1^, (**b**) 10 s^−1^; (**c**) steady-state mobile dislocation density predicted for the of the Ti-17 using “Model G” for different strain rates and temperatures and the comparison with the assumed invariable mobile dislocation density of 10^13^ m^−2^ for the “Model KM”.

The mobile dislocation density is considered constant and independent of temperature and strain rate for the hot deformation modeled using “Model KM”. Thus, the increase in strain rate leads to the same proportional increment in glide velocity in “Model KM” following Orowan’s relationship (Equation (13)). “Model G” predicts an initial near-elastic regime, where the plastic strain rate is negligible (Figure 11a,b). With an increase in stress, the mobile dislocation starts to glide once the thermal stress becomes positive. A peak in the glide velocity and a sharp increase in the plastic strain rate are predicted once the yield stress is reached. The fast multiplication of mobile dislocations follows this peak in the glide velocity. Finally, the reduction in glide velocity and stabilization of the plastic strain rate occurs. A steady-state value of mobile dislocation density, plastic strain rate and mobile dislocation density is reached at very low strains. The sharp increase in mobile dislocation density at the beginning of deformation is responsible for the fast work-hardening predicted by “Model G” (Figure 7) since the predicted mobile dislocation density is higher than the immobile one in “Model G” (Figure 10a,d). Thus, the evolution of the flow stress for “Model G” is related to the mobile dislocation density evolution, with the minor influence of the wall and immobile dislocation density. The differences in mobile dislocation density between 930 °C and 970 °C, and between the different strain rates, are substantially smaller (from ~2 × 10^13^ for 0.001 s^−1^ to ~1 × 10^14^ for 10 s^−1^; Figure 11c) in comparison to the differences in the immobile and wall dislocation densities for the same strain rate difference (Figure 10).

### 4.5. “Model G”: Contributions to Deformation

**Figure 12:**
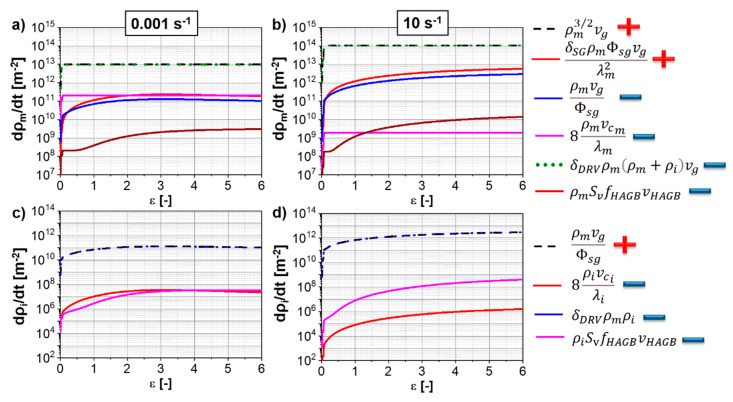
Evolution of the dislocation density reactions for “Model G” for the Ti-17 deformed at 970 °C and (**a**,**c**) 0.001 s^−1^; and (**b**,**d**) 10 s^−1^. (**a**,**b**) mobile dislocation density reactions; (**c**,**d**) immobile dislocation density reactions. The production terms are indicated with a “+” while the deduction ones with a “−“.

One notable difference between “Model KM” and “Model G” is the capability of predicting the contribution of the different deformation phenomena and their impact on the mobile and immobile dislocation density evolutions. Figure 12 shows the evolution of the different dislocation reactions for the mobile (Figure 12a,b) and immobile dislocation density (Figure 12c,d) for Ti-17 deformed at 970 °C. The production rate of mobile dislocations by the Frank–Read source ($\rho_{m}^{3/2}v_{g}$) follows the same kinetics as the annihilation, mainly DRV ($\delta_{DRV}\rho_{m}\left(\rho_{m}+\rho_{i}\right)v_{g}$). The small contributions of the immobilization rate ($\rho_{m}v_{g}/\Phi_{sg}$), SRV due to climb ($8\rho_{m}v_{c}{}_{m}/\lambda_{m}$) and consumption of mobile dislocations due to the movement of HAGBs ($\rho_{m}S_{v}f_{HAGB}v_{HAGB}$) are also predicted.

Annihilation via DRV ($\delta_{DRV}\rho_{m}\rho_{i}$) balances the production of mobile dislocations via the immobilization of mobile ones ($\rho_{m}v_{g}/\Phi_{sg}$) (Figure 12c,d). A minor contribution of the SRV due to climb ($8\rho_{i}v_{c}{}_{i}/\lambda_{i}$) and the consumption of mobile dislocations due to the movement of HAGBs ($\rho_{i}S_{v}f_{HAGB}v_{HAGB}$) are also predicted. A higher strain rate (Figure 12b,d) yields a lower annihilation rate of dislocations via SRV compared to a lower strain rate (Figure 12a,c) for both mobile and immobile dislocation densities. This occurs due to the decrease in time for SRV with the increase in strain rate.

### 4.6. “Model G”: Strain Rates

**Figure 13:**
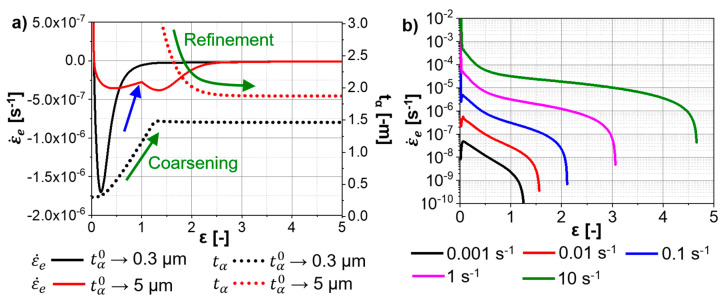
Evolution of the global elastic strain rate predicted by “Model G” for the Ti-17 deformed at (**a**) 810 °C and 0.001 s^−1^, and (**b**) 970 °C. The evolution of the aspect ratio and α-thickness are shown in (**a**) for the initial α-thicknesses of 0.3 µm and 5 µm.

“Model G” predicts elastic and plastic strain rates. The elastic strain rate causes the variation in the stress in each phase following Hook’s law (Equation (16)). Thus, the evolution of the elastic strain rate is an indicator of the hardening/softening behavior; negative elastic strain rates correspond to flow softening, while positive ones to work-hardening. Figure 13a shows the influence of the thickness of the initial α-platelets on the hardening/softening behavior of the Ti-17. An initial hardening that occurs due to the multiplication of dislocations within the α- and β-phases is followed by a softening up to the end of the α-globularization. A more pronounced softening is observed for an initial thickness of 0.3 µm compared to 5 µm. the blue arrow in Figure 13a indicates a change in the kinetics of α-globularization at strain ~1 due to a change in the evolution of the α-globularization once the aspect ratio reaches the value of 1. Figure 13b shows the influence of the strain rate on the hardening kinetics of in Ti-17 deformed at the β-domain. An initial pronounced hardening due to the fast production of mobile dislocations (Figure 11a,b) is followed by a progressive decrease in the hardening. Finally, a slight softening occurs due to the reduction in the factor of strengthening of the wall dislocations when the average boundary misorientation increases. The higher the strain rate, the larger the strain required to achieve this flow softening (Figure 13b).

### 4.7. Predictions for Different Initial Microstructure

**Figure 14:**
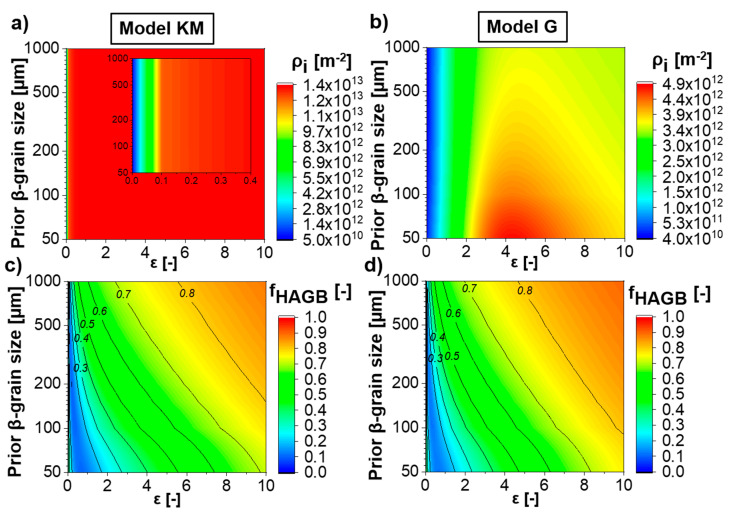
Contour maps of Ti-17 deformed at 830 °C and 0.01 s^−1^ showing the influence of the previous β-grain size on (**a**,**b**) the immobile dislocation density and the (**c**,**d**) fraction of HAGB, predicted by (**a**,**c**) “Model KM” and (**b**,**d**) “Model G”.

The developed models are robust in predicting the evolution of any starting microstructure during deformation. The influence of the previous β-grain size on the immobile dislocation density (Figure 14a,b) and the fraction of HAGB (Figure 14c,d) evolutions for Ti-17 deformed at 830 °C and 0.01 s^−1^ is shown in Figure 14 for both “Model KM” (Figure 14a,c) and “Model G” (Figure 14b,d). The smaller the initial β-grain size, the higher the predicted immobile dislocation density for a given temperature, strain and strain rate (Figure 14b). This occurs due to the higher immobilization rate (Equation (18)) for smaller grain sizes. On the other hand, “Model KM” does not have any term that describes the effect of the boundary density on the production or annihilation of the immobile dislocations. Thus, the previous β-grain size does not influence the immobile dislocation density evolution.

Smaller initial β-grain sizes evolve slower than larger ones (Figure 14c,d). The larger the initial grain size, the faster the increment in boundary average misorientation (Equation (20)). Moreover, smaller grain sizes promote the consumption of the formed low angle grain boundaries due to the movement of high angle grain boundaries (Equation (19)). Therefore, larger initial β-grain sizes lead to a fast evolution of the fraction of high angle grain boundary for a given temperature and strain rate.

### 4.8. Model and Measurements

**Figure 15:**
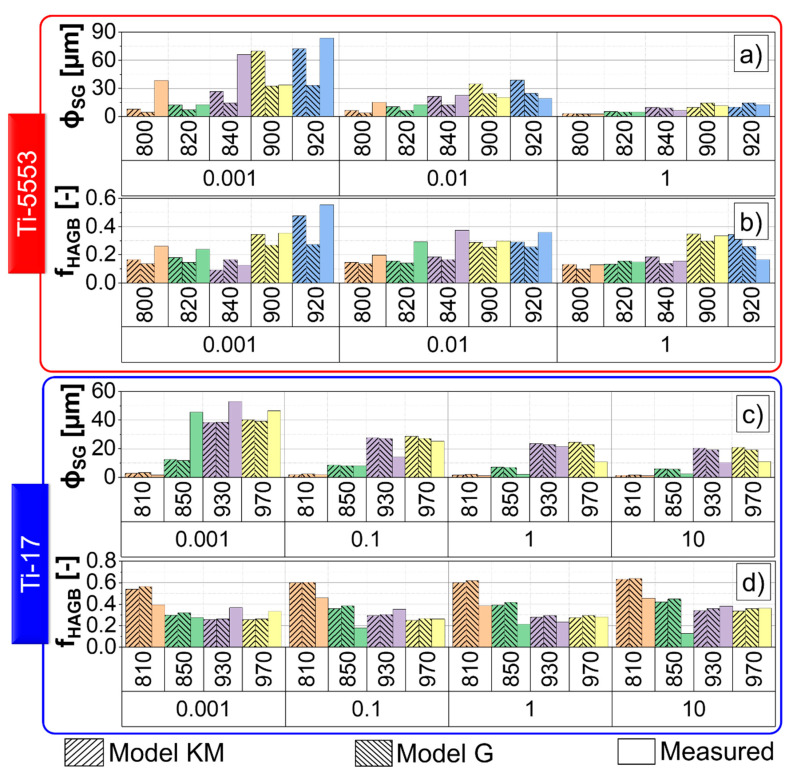
Measured and simulated subgrain size (**a**,**c**) and fraction of HAGBs (**b**,**d**) for the Ti-5553 (**a**,**b**) and Ti-17 (**c**,**d**).

Figure 15 shows the comparison between measured and simulated subgrain size and the fraction of HAGBs for Ti-5553 and Ti-17. The differences between “Model KM” and “Model G” are higher for the Ti-5553, mainly due to the adopted values of $\alpha_{CDRX}$, $\theta_{0}$, $f_{CDRX}$. The simulated and measured values exhibit non-linear behaviors, i.e., an intricate dependency on the strain, strain rate and the temperature is attained. Figure 9 and Figure 10 show that the evolution of the microstructure is a complex function of the input parameters. However, as can also be seen in Figure 14, the developed models are robust in predicting the evolution of any initial microstructure up to any strain, temperature and strain rate.

The differences that are observed can be related to both model assumption and measurements issues.

- **Model assumptions:** the mobile dislocation density is considered constant in “Model KM”, and no term to describe its influence on the cDRX is given. This issue is overcome in “Model G”, but with the assumption that the mobile dislocation is adjusted to yield a constant value once a maximum value is achieved. Additionally, its calculation is performed, assuming a constant thermal stress for a given temperature and strain rate. The limitations on the prediction of mobile dislocation density affect directly both the stress and microstructure evolutions. The effect of texture formation on the thermal stress, or the Taylor factor, is not considered, affecting the overall prediction of the stress evolution, and indirectly the microstructure evolution. Another limitation is the phenomenological description of the HAGB velocity and its power–law dependency uniquely via the strain rate (see Appendix G). This strongly affects the evolution of the boundary density and the average boundary misorientation. In the case of “Model G”, the dependence of the boundary evolution on the applied strain rate is greatly influenced by the phenomenological description of the $\delta_{DRV}$ (see Appendix A), as well as by the constant $f_{CDRX}$ (see Appendix A).
- **Measurements issues**: the limited measurement area size impacts the statistical significance of the measured values. The large previous β-grain size limits the accuracy of the determination of the fraction of HAGBs, since more/less initial β-HAGBs can be considered depending on the measurement site. The formation of the substructure occurs heterogeneously within the β-grain since recovery occurs preferentially in the vicinity of the previous β-HAGBs [58]. Moreover, DRV and consequently cDRX occur differently in each grain, since the Schmid factor of each grain in a polycrystal is different. Thus, the limited measurement area size leads to local information on subgrain size and the fraction of HAGBs that is strongly influenced by the area wherein the EBSD measurement was performed.

## 5. Summary and Conclusions

Two physical models were developed to predict the flow stress and microstructure evolutions of two near-β titanium alloys. A simple dislocation density reaction approach (“Model KM”) is compared to a comprehensive one (“Model G”). cDRX and DRV are the predominant restoration mechanisms. On the other hand, SRV and other phenomena play a negligible role in the restoration of dislocations, as described by “Model G”. The evolution of the mean grain size, the mean subgrain size, the boundary misorientation distribution, and the dislocation densities of all phases can be predicted during deformation. The models can predict the evolution of any initial microstructure. The change in load partitioning mechanism in the α+β-domain is considered to be the reason for the flow softening, and was correlated with the dynamic globularization of the α-phase. The measurements and simulations allow the following conclusions:

- The mobile dislocation density can be estimated by “Model G” for any deformation condition, while it needs to be assumed for the “Model KM”;
- The kinetics of immobile dislocation density evolution depend on the kinetics subgrain size for “Model G”. “Model KM” predicts a fast increase in immobile dislocation density followed by saturation;
- The applied and plastic strain rate are considered the same for “Model KM”, and the calculations start at the yield point. The separation between elastic and plastic strains allows the modeling of the elastic part using “Model G”, as well as the interpretation of the kinetics of hardening and softening in the material;
- Both models predict the dynamic globularization of the α-phase for any initial thickness and aspect ratio, and its evolution is not only described in terms of the fraction of globularization but also the evolution of the microstructural features (thickness, width, the fraction of HAGBs within the α-phase);
- The load partition model describes the overall flow softening in the α+β-domain. The predicted change in the mechanism describes the phenomena related to a decrease in dislocation density, strain rate, and consequently, the stress in the α-phase;
- cDRX is coupled with the dislocation reactions in the same manner for the “Model KM” and “Model G”—recovered dislocations are the source of the formation of new boundaries and increases in their misorientation. However, in “Model G”, only a fraction of the recovered dislocation participates in cDRX;
- The influence of the dislocation reaction approach on cDRX-related variables (subgrain and grain sizes, misorientation distribution, the fraction of HAGBs) is small since the amount of dislocations that contribute to cDRX are comparable between both models;
- “Model G” can be used to predict any dislocation-based phenomena, since the dislocation reactions are defined, and the strain is separated between elastic and plastic.

## Figures and Tables

**Table 1:** Chemical composition of the investigated Ti-5553 and Ti-17 alloys (wt. %).

Alloy	Al	V	Mo	Cr	Sn	Zr	Fe	O	N	C
Ti-5553	5.62	5.03	4.93	2.99	-	-	0.35	0.117	0.0094	0.0075
Ti-17	4.96	0.01	3.91	3.68	1.95	1.91	0.08	0.112	0.05	0.05

**Figure A1:**
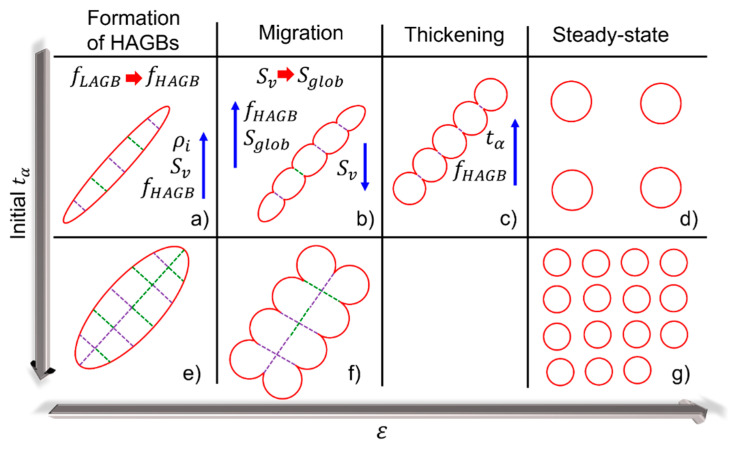
Schematic representation of the dynamic globularization of the α-phase for thin (**a**–**d**) and thicker (**e**–**g**) α-platelets: (**a**,**e**) formation of HAGBs within the α-phase; (**b**,**f**) migration of α/β interfaces though formed boundaries within the α-phase; (**c**) thickening of the formed particles; (**d**,**g**) formation of dispersed and globular α-phase.

## Data Availability

The raw/processed data that support the findings of this study are available from the corresponding author, RHB, upon request.

## Appendix A

The parameters of the initial microstructure were obtained by the EBSD and BSE measurements of the microstructure after annealing and quenching, Table A1.

**Table A1:** Measured microstructural parameters corresponding to the initial microstructure used in the developed model.

		α-Phase		β-Phase	
		Ti-5553	Ti-17	Ti-5553	Ti-17
Initial microstructure	$G_{s}^{0}$ in α+β domain (µm)	3	-	300	500
	$G_{s}^{0}$ in β domain (µm)	-	-	200	500
	$\Phi_{sg}^{0}$ in α+β domain (µm)	3	-		250
	$\Phi_{sg}^{0}$ in β domain (µm)	-	-	200	250
	$t_{\alpha }^{0}$ (µm)	-	3	-	-
	$A_{R}$ (-)	-	15	-	-
	$\bar{\theta_{R}}$ in α+β domain (°)	0.1	0.4	4	0.5
	$\bar{\theta_{R}}$ in β domain (°)	-	-	0.1	0.01

Other parameters are directly obtained from values established in the literature, Table A2.

**Table A2:** Parameters obtained from the literature and used in the developed model.

		α-phase		β-phase	
		Ti-5553	Ti-17	Ti-5553	Ti-17
Parameters obtained from literature	b (nm)	0.295	0.295	0.286	0.286
	M (-)	5	5	3.05	3.05
	Ω (m^3^)	1.7668 × 10^−29^			

Other constants are adjusted according to values established in the literature, Table A3. They are fitted by simultaneously adjusting the flow stress and microstructural results with respect to the measured ones.

**Table A3:** Parameters adjusted according to a range established in the literature and used in the developed model.

			α-Phase		β-Phase	
			Ti-5553	Ti-17	Ti-5553	Ti-17
Parameters fitted from a range established in the literature	$\alpha_{CDRX}$ (-)	Model KM	0.4	0.15	0.2	0.19
		Model G	0.4	0.15	0.2	0.19
	$Q_{HAGB}$ (Jmol^−1^)	Model KM	1.97 × 10^5^	1.97 × 10^5^	1.97 × 10^5^	1.97 × 10^5^
		Model G	1.97 × 10^5^	1.97 × 10^5^	1.97 × 10^5^	1.97 × 10^5^
	n (-)	Model KM	30	15	30	30
		Model G	30	15	30	30
	$\theta_{0}$ (°)	Model KM	1	0.2	0.8	0.7
		Model G	1	0.2	0.8	0.7
	$\theta_{C}$ (°)	Model KM	12	12	12	12
		Model G	12	12	12	12
	${\dot{\varepsilon }}_{ref}$ (s^−1^)	Model KM	1 × 10^6^	1 × 10^6^	1 × 10^6^	1 × 10^6^
		Model G	1 × 10^6^	1 × 10^6^	1 × 10^6^	1 × 10^6^

Other parameters were fitted by simultaneously adjusting the simulated microstructural features with respect to the measured ones, Table A4.

**Table A4:** Fitting parameters used in the developed model.

		α-Phase		β-Phase	
		Ti-5553	Ti-17	Ti-5553	Ti-17
Model KM	$Q_{1}$ (Jmol^−1^)	1.2 × 10^4^	1.2 × 10^4^	1.2 × 10^4^	1.2 × 10^4^
	$Q_{2}$ (Jmol^−1^)	1.2 × 10^4^	1.2 × 10^4^	1.2 × 10^4^	1.2 × 10^4^
	$h_{1}^{0}$ (m^−2^)	Equation (A1)			
	$h_{1}^{KM}$ (m^−2^)	2 × 10^16^	2.2 × 10^16^	4.80 × 10^16^	4 × 10^14^
	$A_{h1}^{Sv}$ (m)	0	1.25 × 10^−2^	3.5 × 10^−3^	1.25 × 10^−2^
	$\eta_{h1}^{KM}$ (-)	-	6.6 × 10^−1^	1	6.6 × 10^−1^
	$h_{2}^{0}$ (-)	Equation (A3)			
	$h_{2}^{KM}$ (-)	3 × 10^−1^	3 × 10^−3^	1.3	1.1 × 10^1^
	$A_{h2}^{Sv}$ (m)	0	1.25 × 10^−2^	3.8 × 10^−3^	1.25 × 10^−2^
	$\eta_{h2}^{KM}$ (-)	-	6.6 × 10^−1^	1	6.6 × 10^−1^
	$E_{MOB}^{0}$ (Wm^−1^)	3.5 × 10^2^	0	6 × 10^1^	2 × 10^4^
	$F_{w}^{0}$ (-)	3 × 10^−2^	1 × 10^−2^	3 × 10^−2^	1 × 10^−2^
	$n_{v}$ (-)	4 × 10^−1^	-	4 × 10^−1^	6.5 × 10^−1^
	$m_{1}$ (-)	3.3 × 10^−1^	8 × 10^−2^	3.3 × 10^−1^	8 × 10^−2^
	$m_{2}$ (-)	3.3 × 10^1^	6 × 10^−1^	3.1 × 10^−1^	8 × 10^−2^
	$A_{glob}$ (-)	-	4 × 10^2^	-	-
	$B_{glob}$ (-)	-	3 × 10^−3^	-	-
	$A_{iso-stress}$ (-)	6 × 10^−2^ (only Ti-5553)			
	$n_{s}$ (-)	3 (only Ti-5553)			
Model G	$W_{g}$ (Jmol^−1^)	2.1 × 10^5^	2.1 × 10^5^	2.1 × 10^5^	2.1 × 10^5^
	$\delta_{DRV}$ (m^−2^)	Equation (A4)			
	$\delta_{DRV}^{0}$ (m^−2^)	6.75 × 10^−7^	3.20 × 10^−7^	5.62 × 10^−7^	3.50 × 10^−7^
	$\eta_{DRV}$ (-)	−1 × 25 × 10^−1^	−6.5 × 10^−2^	−1 × 25 × 10^−1^	−7.75 × 10^−2^
	η (-)	2 × 10^−3^	2 × 10^−3^	2 × 10^−3^	2 × 10^−3^
	$E_{MOB}^{0}\;$(Wm^−1^)	-	-	2.4 × 10^4^	2.4 × 10^4^
	$\eta_{v}$ (-)	-	-	6.5 × 10^−1^	6.5 × 10^−1^
	$f_{CDRX}$ (-)	-	-	Equation (A5)	Equation (A6)
	$f_{CDRX}^{0}$ (-)	2 × 10^−2^	2 × 10^−2^	1.25 × 10^−1^	7.5 × 10^−3^
	$\;f_{gloular}^{interface}$ (m^2^)	-	-	-	1.2 × 10^−10^
	$\;f_{plates}^{interface}$ (m)	-	-	-	2.1 × 10^−5^
	$n_{f_{CDRX}}$ (-)	-	-	-	6.7 × 10^−1^
	$A_{glob}$ (-)	-	4 × 10^2^	-	-
	$B_{glob}$ (-)	-	3 × 10^−2^	-	-

Some parameters are empirically adjusted according to expressions. The $h_{1}^{0}$ is given by Equation (A1) to consider the role of the α/β interface density ($S_{interface}$, Equation (A2)) in the production of immobile dislocations.

(A1) $$h_{1}^{0}=\left(1+{\left(A_{h1}^{Sv}S_{interface}\right)}^{\eta_{h1}^{KM}}\right)h_{1}^{KM}$$

(A2) $$S_{interface}=\{\begin{array}{c} \begin{array}{cc} \frac{2F_{v}}{t_{\alpha }^{0}}, & Ti-17 \end{array}\\ \begin{array}{cc} \frac{3F_{v}}{r_{\alpha }^{0}}, & Ti-5553 \end{array} \end{array}$$

$r_{\alpha }^{0}$ is the mean radius of the α-particle in Ti-5553 and is equal to $G_{s}^{0}$. The $h_{2}^{0}$ is also given as a function of the α/β interface density; Equation (A3).

(A3) $$h_{2}^{0}=\left(1+{\left(A_{h2}^{Sv}S_{interface}\right)}^{\eta_{h2}^{KM}}\right)h_{2}^{KM}$$

The $\delta_{DRV}$ is given as a power–law function of the plastic strain rate; Equation (A4).

(A4) $$\delta_{DRV}=\delta_{DRV}^{0}{\left(\frac{{\dot{\varepsilon }}_{p}}{{\dot{\varepsilon }}_{ref}}\right)}^{\eta_{DRV}}$$

The $f_{CDRX}$ is given as a function of the $S_{interface}$ and is given by Equations (A5) and (A6) for the Ti-5553 and Ti-17, respectively.

(A5) $$f_{CDRX}\;=\;f_{CDRX}^{0}+\;\frac{\;f_{gloular}^{interface}S_{interface}}{\lambda_{interspace}},\;\;\;\;Ti-5553$$

(A6) $$f_{CDRX}\;=\;f_{CDRX}^{0}+\;{\left(\;f_{plates}^{interface}S_{interface}\;\right)}^{n_{f_{CDRX}}},\;\;\;\;Ti-17$$

$\lambda_{interspace}$ is the particle interspace and is calculated according to Equation (A7) [58] assuming a random distribution of spherical α-phase for the Ti-5553.

(A7) $$\lambda_{interspace}\;=\sqrt{\frac{\ln\left(3\right)}{2\pi n_{d}R_{\alpha }^{0}}+\frac{8R_{\alpha }^{0}{}^{2}}{3}}-\sqrt{\frac{8}{3}}R_{\alpha }^{0},\;\;\;\;n_{d}=\;\frac{3f_{\alpha }}{4\pi {\left(R_{\alpha }^{0}\right)}^{3}}$$

## Appendix B

The initial subgrain ($\Phi_{sg}^{0}$) and grain ($G_{s}^{0}$) sizes, average boundary misorientation angle and dislocation densities define the initial microstructure. Equation (A8) [46] describes the maximum boundary surface density that is given by the total surface of boundaries (HAGB + LAGB) over the total volume.

(A8) $$S_{v}^{0}=\frac{2}{\Phi_{sg}^{0}}$$

Equation (A9) describes the HAGB surface density corresponding to the initial grain size.

(A9) $$S_{v_{HAGB}}^{0}=\frac{2}{G_{s}^{0}}$$

Equation (A10) describes the initial boundary surface density prone to evolve during the deformation.

(A10) $$S_{v_{CDRX}}^{0}=S_{v}^{0}-S_{v_{HAGB}}^{0}$$

For an initial fully recrystallized microstructure, $S_{v}^{0}≅S_{v_{HAGB}}^{0}$. Finally, a multiplicative factor $A_{1}^{0}$ that represents the fraction of boundaries prone to evolve in misorientation during the deformation is calculated using Equation (A11).

(A11) $$A_{1}^{0}=\frac{S_{v_{CDRX}}^{0}}{S_{v}^{0}}$$

The misorientation ranges given by the Rayleigh distribution, and the Mackenzie distributions range between 0 and 60.7° (≅1.06 rad) [50]. Equation (A12) represents the initial fraction of Rayleigh distribution, $f_{R}^{0}$.

(A12) $$f_{R}^{0}=\overset{60.7}{\underset{0}{\int }}\Theta_{R}^{0}\left(\theta \right)d\theta$$

The fraction ($1-A_{1}^{0}f_{R}^{0}$) describes the boundary misorientation angle distribution with a Mackenzie distribution. Equation (A13) describes the initial boundary misorientation distribution.

(A13) $$\Theta^{0}\left(\theta \right)=A_{1}^{0}f_{R}^{0}\Theta_{R}^{0}\left(\theta \right)+\left(1-A_{1}^{0}f_{R}^{0}\right)\Theta_{M}^{0}\left(\theta \right)$$

If the initial microstructure is fully recrystallized, $A_{1}^{0}$ = $f_{R}^{0}$ = 0, and the boundary misorientation follows a Mackenzie distribution, while the boundary misorientation distribution of an initial microstructure with a high fraction of LAGB is mainly represented by a Rayleigh distribution ($A_{1}^{0}\approx$ $f_{R}^{0}$ ≈ 1).

The initial wall dislocation density ($\rho_{w}^{0}$) is calculated according to Equation (9).

(A14) $$\rho_{w}^{0}=\frac{nS_{v}^{0}{\bar{\theta }}_{LAGB}^{0}f_{LAGB}^{0}}{b}$$

${\bar{\theta }}_{LAGB}^{0}$ is the mean misorientation of the boundary distribution, and $f_{LAGB}^{0}$ is the initial fraction of LAGBs and is given according to Equation (A15).

(A15) $$f_{LAGB}^{0}=\int_{0}^{12}\Theta^{0}\left(\theta \right)d\theta$$

The value 12° (≅0.21 rad) is the transition angle between an LAGB and an HAGB.

## Appendix C

Non-sharp LAGBs contributes to a higher internal athermal stress in comparison to sharp LAGBs (considered negligible [18]). An empirical factor ($F_{w}$) gives the strain field size caused by a non-sharp LAGB (Equation (A16)).

(A16) $$F_{w_{x}}=F_{w_{x}}^{0}\int_{0}^{3}\Theta_{x}\left(\theta \right)d\theta ,\;\;\;\;x=\;\alpha ,\;\beta$$

The transition angle of 3° (≅0.05 rad) describes the transition between non-sharp and sharp LAGBs. When the substructure is forming, $\bar{\theta }$ < 3°, and the contribution of the non-sharp LAGBs is higher. An increase in boundary misorientations decreases the $F_{w}$ value asymptotically to zero.

## Appendix D

A simple approach is considered to calculate the yield stress assuming an Arrhenius-type behavior (Equation (A17)) [59].

(A17) σYS=1αYSln{(ε˙exp(QYSRT)AYS)1nYS+[(ε˙exp(QYSRT)AYS)12nYS+1]12}

where $\dot{\varepsilon }$ is the strain rate, R is the universal constant of gases (8.314 J.mol^−1^.K^−1^) and T the temperature. The values of the activation energy ($Q_{YS}$), $\alpha_{YS}$, $A_{YS}$, and $n_{YS}$ that define the yield stress of the α- and β-phases are listed in Table A5. A detailed procedure for the parameter calculation was published elsewhere [59]. For both alloys, the parameters of the yield stress of the β-phase are obtained by fitting the measured yield stress in the β-domain with Equation (A17). The yield stress of the β-phase is then extrapolated from the values obtained for the α+β-domain. The yield stress of the α-phase is estimated based on the yield stress values in the α+β-domain using a simple law of mixtures for the fractions of α- and β-phases. Finally, the parameters of the yield stress of the α-phase are obtained by fitting with Equation (A17) the estimated yield stress of the α-phase in the α+β-domain.

**Table A5:** Constants used for the calculation of the yield stress.

	α-Phase		β-Phase	
	Ti-5553	Ti-17	Ti-5553	Ti-17
$Q_{YS}$ (Jmol^−1^)	1.58 × 10^5^	1.58 × 10^5^	2.38 × 10^5^	2.38 × 10^5^
$\alpha_{YS}$ (MPa^−1^)	2.85 × 10^−3^	2.80 × 10^−3^	1.16 × 10^−2^	1.85 × 10^−2^
$A_{YS}$ (s^−1^)	2.22 × 10^6^	1.78 × 10^6^	2.17 × 10^9^	1.80 × 10^6^
$n_{YS}$ (-)	3.33	4.24	3.23	2.94

## Appendix E

In the iso-strain regime, Equation (A18) is fulfilled:

(A18) $$\dot{\varepsilon }={\dot{\varepsilon }}_{\alpha }^{\varepsilon }={\dot{\varepsilon }}_{\beta }^{\varepsilon },$$

$\dot{\varepsilon }$, ${\dot{\varepsilon }}_{\alpha }^{\varepsilon }$ and ${\dot{\varepsilon }}_{\beta }^{\varepsilon }$ are the applied strain rate, the strain rate in the α-phase, and the strain rate in the β-phase, respectively.

Equations (A19) and (A20) describe the iso-power model [55].

(A19) $$\dot{\varepsilon }=F_{v}{\dot{\varepsilon }}_{\alpha }^{W}+\left(1-F_{v}\right){\dot{\varepsilon }}_{\beta }^{W}$$

(A20) $$\sigma_{\alpha }{\dot{\varepsilon }}_{\alpha }^{W}=\sigma_{\beta }{\dot{\varepsilon }}_{\beta }^{W}$$

${\dot{\varepsilon }}_{\alpha }^{W}$ and ${\dot{\varepsilon }}_{\beta }^{W}$ are strain rates in the iso-power condition of the α- and β-phases, respectively. $F_{v}$ is the fraction of the α-phase.

For the iso-stress regime, the flow stress of the β-phase is equal to the flow stress for the α-phase. The strain rates in the α- and β-phases are calculated by Equation (A21) [59] and Equation (A22), respectively.

(A21) $${\dot{\varepsilon }}_{\alpha }^{\sigma }=\exp\left(n_{YS_{\alpha }}\ln\left(\sinh\left(\alpha_{YS_{\alpha }}\sigma_{\beta }\right)\right)-\frac{Q_{YS_{\alpha }}}{RT}+\ln\left(A_{YS_{\alpha }}\right)\right)$$

(A22) $${\dot{\varepsilon }}_{\beta }^{\sigma }=\frac{F_{v}{\dot{\varepsilon }}_{\alpha }^{\sigma }-\dot{\varepsilon }}{\left(1-F_{v}\right)}$$

## Appendix F

Figure A1 shows the process of dynamic α-globularization schematically. The migration of the formed and existing interfaces promoting the thickening of the α-platelet accompanies the formation of new α/β interfaces. The movement of the α/β interfaces consuming the existing HAGBs within the α-phase occurs with a velocity, $v_{glob}$, that is empirically correlated with the glide velocity in the β-phase by a constant and fitting parameter of the model $A_{glob}$ (Equation (A23)).

(A23) $$v_{glob}=A_{glob}v_{g_{\beta }}$$

Semiatin et al. [60] proved that the growth of the spherical particles during dynamic globularization is a faster Ostwald ripening process driven by pipe diffusion. During the Ostwald ripening of non-spherical particles [61], complex solutions are necessary. Here, the growth of the α-particles is simplified assuming an empirical velocity of growth, $v_{growth}$, related to the climb velocity of the mobile dislocation density in the β-phase by a constant and fitting parameter of the model $B_{glob}$ (Equation (A24)).

(A24) $$v_{growth}=\;B_{glob}v_{climb_{\beta }}$$

The evolution of the morphological features in the α-phase depends on the aspect ratio of the formed particles:

- Aspect ratio larger than one:

Equations (A25)–(A27)describe the thickness evolution rate, the width evolution rate, and the aspect ratio, respectively.

(A25) $$\frac{dt_{\alpha }}{dt}=2f_{glob}v_{growth}$$

(A26) $$\frac{dw_{\alpha }}{dt}=-A_{R}{}^{2}t_{\alpha }^{3}\frac{dt_{\alpha }}{dt}$$

(A27) $$A_{R}=\frac{2w_{\alpha }^{2}}{t_{\alpha }\left(S_{glob}w_{\alpha }^{2}+2w_{\alpha }\right)}$$

Equations F3 to F5 are used until the aspect ratio is larger than one. Even if $f_{glob}$ = 1, the aspect ratio is not necessarily one. A small α-thickness can lead to the consumption of the formed boundaries within the α-phase quickly, and the α-globularization ends with an aspect ratio larger than one (Figure A1b). This is followed by thickening (Figure A1c) until reaching the steady state (Figure A1d).

- Aspect ratio equals to one:

Equation (A28) describes the thickness update for the iteration i+1 ($t_{\alpha }^{i+1}$) as a function of the density of the α/β interface formed during dynamic globularization of the α-phase ($S_{glob}$) and the thickness from the iteration i ($t_{\alpha }^{i}$).

(A28) $$t_{\alpha }^{i+1}\;=\frac{4t_{\alpha }^{i}}{S_{glob}t_{\alpha }^{i}\;+\;4t_{\alpha }^{i}}$$

A large initial thickness of the α-platelet leads to their division into particles of smaller thickness than the initial one (Figure A1f). This occurs when the aspect ratio reaches one while α-globularization fraction is still not finished.

## Appendix G

Equation (A29) [46] describes phenomenologically the velocity of HAGBs, $v_{HAGB}$.

(A29) $$v_{HAGB_{x}}=v_{0_{x}}{\left(\frac{{\dot{\varepsilon }}_{p_{x}}}{{\dot{\varepsilon }}_{ref}}\right)}^{n_{v_{x}}}\exp\left(-\frac{Q_{HAGB_{x}}}{RT}\right),\;\;\;\;x=\;\alpha ,\;\beta$$

$v_{0}$ is a coefficient, $n_{v}$ is an exponent and $Q_{HAGB}$ is the activation energy for the movement of HAGBs.

## Appendix H

Equations (A30) and (A31) [46] describe the hardening and recovery coefficients, respectively.

(A30) $$h_{1}{}_{x}=h_{1_{x}}^{0}{\left(\frac{{\dot{\varepsilon }}_{x}}{{\dot{\varepsilon }}_{ref}}\right)}^{m_{1}{}_{x}}\exp\left(\frac{Q_{1}{}_{x}}{RT}\right),\;\;\;\;x=\;\alpha ,\;\beta$$

(A31) $$h_{2}{}_{x}=h_{2}^{0}{}_{x}{\left(\frac{{\dot{\varepsilon }}_{x}}{{\dot{\varepsilon }}_{ref}}\right)}^{-m_{2}{}_{x}}\exp\left(-\frac{Q_{2}{}_{x}}{RT}\right),\;\;\;\;x=\;\alpha ,\;\beta$$

where $h_{1}^{0}$ and $h_{2}^{0}$ are the constants for the production of dislocations and dynamic recovery coefficients, respectively; $m_{1}$ and $\;m_{2}$ are exponents related to the strain hardening coefficient; $Q_{1}$ and $Q_{2}$ are activation energies for the production of dislocations and DRV, respectively.
